# The Correlation between Epicardial Adipose Tissue Thickness Measured by Echocardiography and P-Wave Dispersion and Atrial Fibrillation

**DOI:** 10.31083/j.rcm2508287

**Published:** 2024-08-15

**Authors:** Qing-xue Zhang, Zhi-jian Liu, Xiao-hong Liu, Xiao-hui Zhao, Xiu-chang Li

**Affiliations:** ^1^Department of Echocardiography, The Second Affiliated Hospital of Shandong First Medical University, 271000 Taian, Shandong, China; ^2^Department of Cardiology, The Second Affiliated Hospital of Shandong First Medical University, 271000 Taian, Shandong, China

**Keywords:** echocardiography, epicardial adipose tissue, atrial fibrillation, P-wave dispersion

## Abstract

**Background:**

Recent studies have indicated a close relationship between the thickness of epicardial adipose tissue (EAT) and the occurrence as well as persistence of atrial fibrillation (AF). However, the pathogenesis of this association is still in the exploratory stage. The aim of this study is to explore the correlation EAT, as measured by echocardiography, and P-wave dispersion (Pd) in the context of atrial fibrillation. Additionally, the study seeks to analyze the utility of EAT at different anatomical sites in identifying individuals who are predisposed to atrial fibrillation.

**Methods:**

A total of 136 subjects were enrolled and categorized into groups based on the guidelines: paroxysmal atrial fibrillation group (PAF group), persistent atrial fibrillation group (AF group), and non-atrial fibrillation group. Comprehensive clinical data, including general information and medications that could impact the occurrence of atrial fibrillation, were gathered for all patients. Echocardiography was employed to measure the maximum EAT thickness near the apex of the heart on the anterior right ventricular wall and near the base of the right ventricle for each participant. Pd values were computed for each patient based on standard 12-lead synchronous electrocardiogram (ECG). The study involved comparing the disparity in EAT thickness between the two specified sites across the three groups. Additionally, correlation analyses were performed to assess the relationship between EAT thickness at the two sites and Pd. Regression analysis was applied to explore potential risk factors for atrial fibrillation. The diagnostic value of EAT at each site in predicting atrial fibrillation was evaluated using Receiver Operating Characteristic curve (ROC) analysis.

**Results:**

EAT thickness of the anterior wall near the apex of the heart and near the base of the right ventricle were significantly positively correlated with Pd (*p* < 0.05), EAT thickness near the base and left atrial diameter were independent risk factors for atrial fibrillation (OR = 13.673, 95% CI 2.819~66.316, *p* = 0.001; OR = 2.294, 95% CI 1.020~5.156, *p* = 0.045). ROC analysis showed that the area under the curve of EAT thickness near the heart base was 0.723, and the best threshold for predicting the occurrence of AF was 1.05 cm.

**Conclusions:**

The echocardiography-measured epicardial adipose tissue thickness, particularly in proximity to the heart base, exhibits a significant correlation with Pd. Notably, EAT thickness near the heart base demonstrates superior predictive capability for atrial fibrillation compared to thickness near the apex.

## 1. Introduction

Some studies have found that epicardial adipose tissue (EAT) is significantly correlated with the occurrence and maintenance of atrial fibrillation (AF) [1, 2, 3]. AF is a very common arrhythmia in clinical practice, with a high morbidity, disability and mortality rate around the world. Although the mechanism of AF is still being explored, more and more studies have proved that atrial electrical remodeling plays a key role in the occurrence and development of AF [4]. Among many clinical cardiac electrophysiological indicators, electrocardiogram (ECG) P-wave, which represents the comprehensive vector of atrial depolarization process, can best reflect the atrial electrical activity [5]. Recent result had shown that the P-wave indicators of 12-lead ECG, especially the P-wave dispersion (Pd) [6] could be used to early screen the patients with paroxysmal AF.

Although many studies have explored the relationship between EAT and AF, the correlation between EAT thickness and Pd was not reported. Is the relationship between EAT and atrial fibrillation related to its effect on Pd. In addition, since the local endocrine effect of EAT plays a significant role in the occurrence and development of cardiovascular diseases, does the thickness of fat in different sites of pericardium have different influences on the occurrence of AF?

## 2. Materials and Methods

### 2.1 Study Objects

This study included a total of 136 patients, comprising 30 individuals with persistent AF (18 males and 12 females, aged 42–88 years), 30 patients with paroxysmal AF (13 males and 17 females, aged 44–83 years), and 76 patients with normal sinus rhythm (43 males and 33 females, aged 31–74 years). General clinical data, such as gender, age, blood pressure, diabetes and coronary heart disease, and previous clinical medication that may affect the occurrence of AF were collected. All the subjects had clear and complete echocardiographic images and 12-lead synchronous ECG images, and had clear clinical diagnosis. Underlying diseases included coronary heart disease, hypertension, or diabetes. This study obtained the informed consent of the patients and was approved by the medical ethics committee of the Second Affiliated Hospital of Shandong First Medical University (Approval number: 2021-099).

Patients who met the following conditions were excluded from this study: (1) Left Ventricular Ejection Fraction (LVEF) less than 50%; (2) Patients with moderate or severe valvular heart disease, congenital heart disease, hypertrophic cardiomyopathy and history of myocardial infarction; (3) Patients who had undergone radiofrequency ablation of AF, pacemaker implantation, or artificial heart valve; (4) Combined with other serious malignant wasting diseases (such as malignant tumors); (5) Those with hyperthyroidism and/or severe hepatic and renal insufficiency; (6) Those with poor ultrasound and/or ECG image quality.

### 2.2 Methods

#### 2.2.1 Methods of Echocardiography

**Fig. 1.**
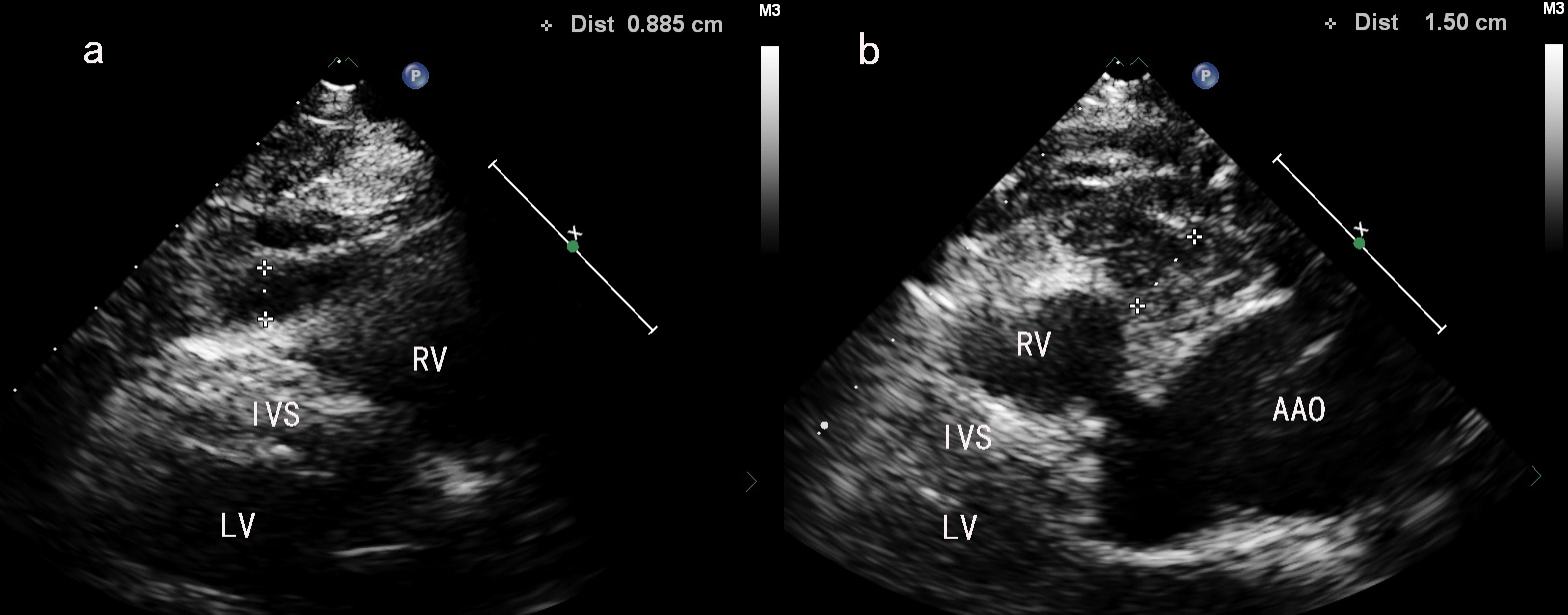
**Measurement of epicardial adipose tissue (EAT) thickness in different sites of in front of the anterior wall of the right ventricle**. (a) Schematic diagram of measuring EAT_Apex_ in PLAX view. (b) Schematic diagram of measuring EAT_Basal_ in PLAX view. PLAX, parasternal long-axis view; RV, right ventricle; LV, left ventricle; IVS, interventricular septum; AAO, ascending aorta.

Philips Epiq7c color Doppler system equipped with the phased array probe S5-1 (1~5 MHz) echocardiography system (Philips, Amsterdam, Netherland) was used to perform the echo examination. The parasternal long-axis view of the left ventricle was obtained. At the end of systole, the outer edge of the myocardium of the anterior wall of the right ventricle and the inner edge of the visceral layer of the pericardium were used as the anterior and posterior boundary, respectively, and the relative hypoechoic area was measured vertically (Fig. 1a,b) as the EAT. The EAT near the apex was defined as EAT_Apex_ and the EAT near the heart base was defined as EAT_Basal_. For each patient, a standardized procedure was followed, involving the routine selection of three cardiac cycles, with measurements taken three times to derive an average value. Left ventricular ejection fraction (LVEF, measured by biplane Simpson method), left ventricular diameter, mitral flow spectrum E peak and mitral annulus tissue Doppler e’ peak were measured, and E/e’ was calculated. Data measurements for each patient during all echocardiographic examinations were determined by the same senior physician.

#### 2.2.2 Methods of Electrocardiogram

A 12-lead synchronous ECG was obtained and the P-wave duration of all leads in the same cardiac cycle was measured. The difference value of maximum and minimum P-wave duration was defined as the Pd [6]. Briefly, a 12-lead synchronized ECG was recorded with a paper walking speed of 25 mm/s and an amplitude of 10 mm/mv. The cardiac cycle in which the image was clear and stable was selected on the ECG workstation (Smart ECG net, EDAN Instruments, Inc., China), and the image was magnified for measurement. The first intersection point of the P wave with the baseline was set as the starting point of the measurement, and the second intersection point with the baseline was set as the end point of the measurement. After the P-wave duration were measured in all 12 leads of the same cardiac cycle, the P wave dispersion is derived by subtracting the minimum P wave duration from the maximum in any of the 12 ECG leads.

### 2.3 Other Indicators

The primary comorbid conditions such as coronary heart disease, hypertension, diabetes, etc., were documented for each group. Additionally, prior clinical use of drugs, with a particular focus on calcium channel blockers and beta-receptor blockers, which could potentially influence the development of AF, was recorded.

### 2.4 Statistics Processing

Measurement data are represented as ($\bar{\chi }$ ± s), and K-S test was used as a normality test. If the measurement data conformed to normal distribution, a *t* test was used for comparison between two groups, analysis of variance (ANOVA) was used for comparison between multiple groups, and the least significant difference (LSD) method was used for pairwise comparison. Categorical data are presented as cases (n) or percentages (%), and the Chi-square test was employed for statistical analysis. Pearson correlation analysis was utilized to investigate the correlation between EAT thickness and Pd values. Logistic regression was employed to analyze potential risk factors contributing to AF. Receiver Operating Characteristic (ROC) analysis was conducted to assess the predictive value of EAT thickness measured at two distinct sites for the occurrence of AF. A value of *p* < 0.05 was considered statistically significant.

## 3. Results

### 3.1 General Clinical Data Analysis

A total of 136 patients were enrolled into this study, including 74 males and 62 females, aged 31–88 years. And the patients were divided as AF group (n = 30), paroxysmal AF (PAF) group (n = 30) and non-AF group (n = 76). Comorbidities included coronary heart disease, hypertension or diabetes, and some patients had two or three conditions. There were no significant differences in age, gender, concomitant diseases and the rate of previous clinical medication that may affect the occurrence of AF among the three groups (*p* > 0.05 for all). The left atrial diameter (LAD) and E/e’ values exhibited significant differences among the three groups (*p* < 0.05). Subsequent LSD testing revealed that the AF group had a significantly larger left atrial diameter compared to both the PAF group and non-AF group (*p* = 0.04 and *p* < 0.001, respectively), while no significant difference was observed between the PAF group and non-AF group (*p* > 0.05). Additionally, LSD testing of E/e’ values demonstrated a significant difference between the non-AF group and AF group (*p* = 0.011), whereas no significant difference was found between the PAF group and any other groups (*p* > 0.05). The results of the general clinical data analysis of patients in the above groups are shown in Table 1.

**Table 1.** **Comparison of general clinical data in each group**.

	AF (n = 30)	PAF (n = 30)	non-AF (n = 76)	*F/χ^2^*	*p*
Age (year, $\bar{\chi }$ ± s)	62.97 ± 10.72	60.53 ± 9.18	59.47 ± 9.35	1.420	0.246
Gender (n, M/F)	18/12	13/17	43/33	2.000	0.367
BMI ($\bar{\chi }$ ± s)	25.03 ± 2.68	25.93 ± 4.73	26.59 ± 3.42	2.043	0.134
Coronary heart disease (%)	13 (43.33)	12 (40.00)	33 (43.42)	0.110	0.946
Hypertension (%)	19 (63.33)	18 (60.00)	44 (55.36)	0.633	0.729
Diabetes (%)	8 (26.67)	8 (26.67)	17 (22.37)	0.337	0.845
Use rate of calcium channel blockers (%)	43.33	40.00	50.00	1.003	0.605
Use rate of β-blocker (%)	46.67	40.00	44.73	0.297	0.862
Use rate of metformin (%)	13.33	6.67	15.79	1.559	0.459
Use rate of SGLT2i (%)	20.00	13.33	10.53	1.681	0.431
Use rate of statin (%)	26.67	20.00	31.58	1.463	0.481
E/e’ ($\bar{\chi }$ ± s)	13.36 ± 4.82	11.57 ± 3.94	11.05 ± 4.00	3.302	0.040

n, sample size; AF, atrial fibrillation; PAF, paroxysmal atrial fibrillation; M, male; F, female; BMI, body mass index; SGLT2i, sodium-glucose cotransporter 2 inhibitors; E/e’, the ratio between early mitral inflow velocity and mitral annular early diastolic velocity.

### 3.2 Comparison of EAT in Different Groups and Different Sites

The student *t*-test revealed a significant difference between EAT_Basal_ and EAT_Apex_, where EAT_Basal_ (1.016 ± 0.30 cm) was significantly higher than EAT_Apex_ (0.727 ± 0.23, *p* < 0.001). ANOVA analysis demonstrated a significant difference in both EAT_Apex_ and EAT_Basal_ among the three groups (EAT_Apex_: *p* = 0.001, EAT_Basal_: *p* < 0.001). The LSD test results indicated that compared to the non-AF group, both EAT_Apex_ and EAT_Basal_ values were significantly higher in the AF and PAF groups (*p* < 0.05 for all), while the AF group had significantly higher levels of EAT_Basal_ compared to the PAF group (*p* = 0.001). However, there was no statistical difference was observed in terms of EAT_Apex_ between the AF and PAF groups (*p* = 0.181, Table 2).

**Table 2.** **Comparison of EAT values of different sites**.

	AF (n = 30)	PAF (n = 30)	non-AF (n = 76)	*F*	*p*
EAT_Apex_ (cm, $\bar{\chi }$ ± s)	0.84 ± 0.29	0.77 ± 0.23	0.67 ± 0.17	7.735	0.001
EAT_Basal_ (cm, $\bar{\chi }$ ± s)	1.26 ± 0.30	1.03 ± 0.37	0.91 ± 0.20	18.563	<0.001

n, sample size; AF, atrial fibrillation; PAF, paroxysmal atrial fibrillation; EAT, epicardial adipose tissue.

### 3.3 Correlation Analysis of EAT and Pd Value

**Fig. 2.**
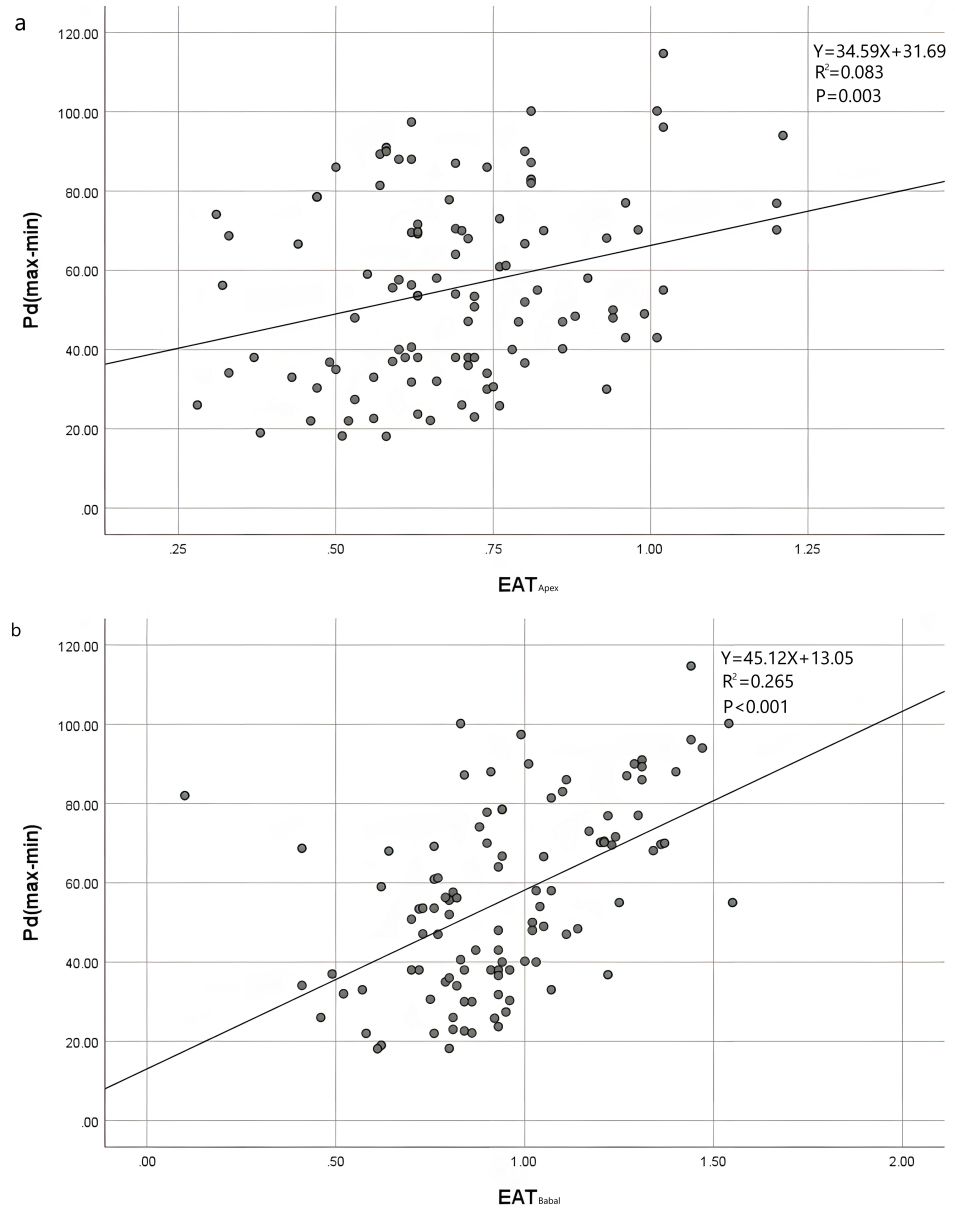
**Scatter plots of the thickness of EAT thickness and Pdin different sites in front of the anterior wall of the right ventricle**. (a) Scatter plot of correlation between EAT_Apex_ and Pd. (b) Scatter plot of correlation between EAT_Basal_ and Pd. EAT, epicardial adipose tissue; Pd, P-wave dispersion.

The data from both the PAF group and non-AF group were combined, and Pearson correlation analysis was conducted to examine the associations between EAT_Apex_ and Pd, as well as EAT_Basal_ and Pd, and Scatter plots were generated (Fig. 2a,b). The findings demonstrated a significant positive correlation between EAT_Basal_ and Pd values (r = 0.515, *p* < 0.01). Additionally, a weak positive correlation was observed between EAT_Apex_ and Pd values (r = 0.288, *p* = 0.03).

### 3.4 Risk Factors Analysis of Atrial Fibrillation

The patients were categorized into two groups: an AF group (including both AF and PAF subgroups) and a non-AF group. The occurrence of AF was considered as the dependent variable, while potential risk factors for AF such as gender, age, coronary heart disease, hypertension, diabetes, LAD, LVEF, E/e’, and EAT thickness were set as independent variables. Logistic regression analysis revealed that LAD and EAT_Basal_ independently contributed to the risk of AF (OR = 2.294; 95% CI 1.020–5.156; *p* = 0.045; OR = 13.673; 95% CI 2.819–66.318; *p* = 0.01). Detailed results are presented in Table 3.

**Table 3.** **Risk factors of AF**.

	*p*	OR	95% CI
LAD (>40 mm)	0.045	2.294	1.020∼5.156
Gender	0.461	0.738	0.329∼1.656
Age	0.576	1.013	0.969∼1.059
Coronary heart disease	0.589	1.244	0.563∼2.750
Hypertension	0.599	0.801	0.350∼1.832
Diabetes	0.940	0.964	0.372∼2.497
E/e’	0.534	1.296	0.572∼2.941
EAT_A_	0.175	4.780	0.499∼45.815
EAT_B_	0.001	13.673	2.819∼66.318

LAD, left atrial diameter; EAT, epicardial adipose tissue; OR, odds ratio; CI, confidence interval; AF, atrial fibrillation; E/e’, the ratio between early mitral inflow velocity and mitral annular early diastolic velocity.

### 3.5 Value of EAT in Predicting the Occurrence of Atrial Fibrillation

**Fig. 3.**
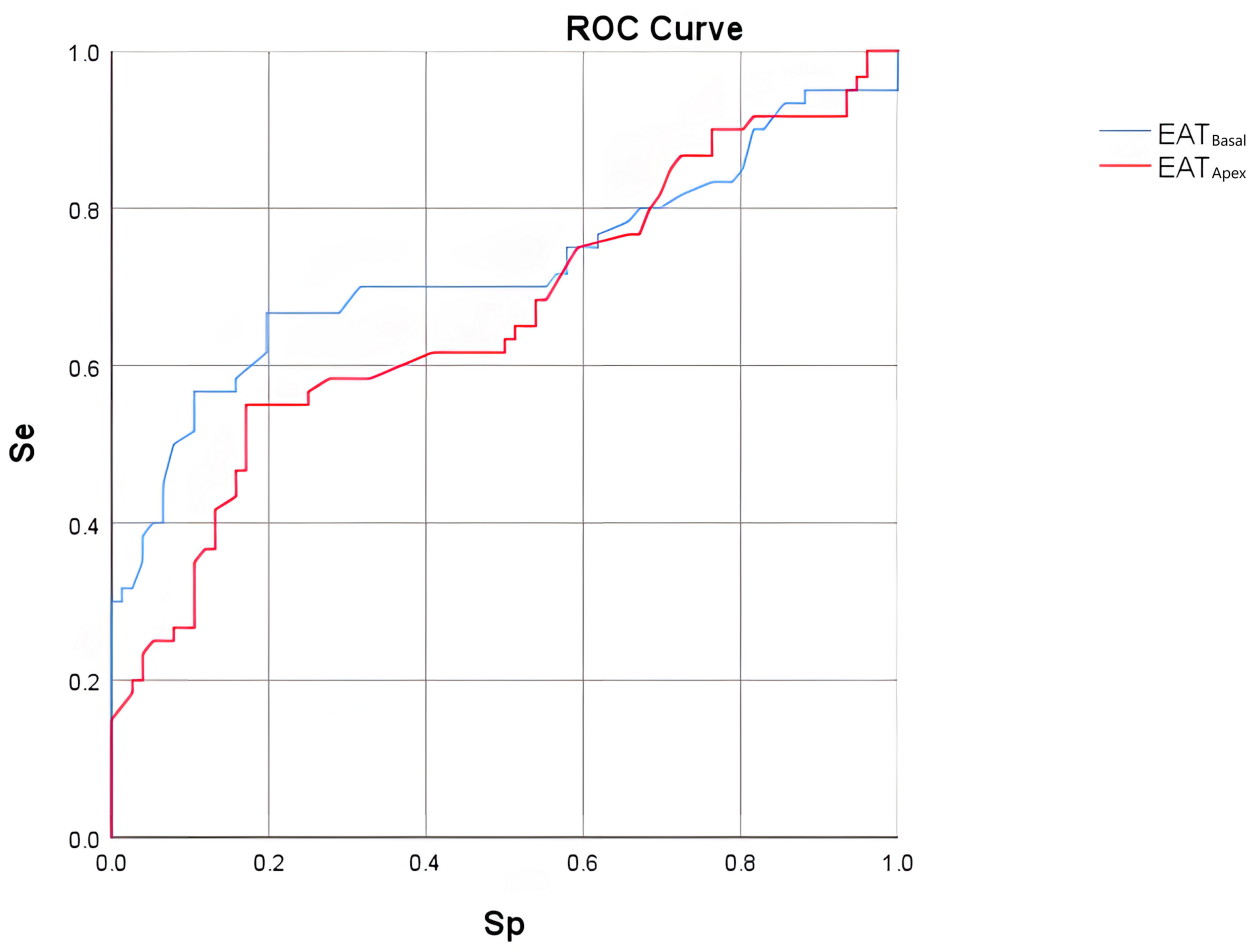
**ROC curve analysis of EAT_𝐀𝐩𝐞𝐱_ and EAT_𝐁𝐚𝐬𝐚𝐥_ in predicting AF occurrence**. ROC, receiver operating characteristic; EAT, epicardial adipose tissue; AF, atrial fibrillation; Se, sensitivity; Sp, specificity.

To assess the predictive value of EAT in AF, we generated receiver operating characteristic curves for EAT thickness at two sites to predict AF occurrence (including paroxysmal and persistent AF). The area under the curve for EAT_Basal_ was 0.723, with a threshold of 1.05 cm providing optimal prediction accuracy: sensitivity was 66.67% and specificity was 80.26% (Fig. 3). In contrast, EAT_Apex_ had a lower predictive power, with an area under the curve of only 0.665.

## 4. Discussion

It is widely accepted that AF arises from the presence of multiple reentry waves conducting, disappearing or splitting within the atrial tissue. Prolonged AF gradually induces structural changes in the heart, ultimately leading to adverse cardiac outcomes such as stroke and heart failure [7]. The occurrence and persistence of AF are influenced by various factors. Recent studies have suggested a potential association between EAT and the development of AF [8, 9, 10]. Some studies have demonstrated a linear correlation between increased EAT quantity and elevated risk of AF (95% CI: 1.22~1.43, *p* < 0.01) [11], with increased EAT volume identified as an independent risk factor for this condition [12]. Our study also observed significantly higher levels of EAT in both the AF group and PAF group compared to the non-AF group, with patients experiencing longer durations of AF exhibiting more pronounced increases in EAT deposition. These findings support the hypothesis that elevated EAT may contribute to an augmented risk for developing and sustaining AF.

In our investigation, we noted an uneven distribution pattern of epicardial fat deposition. Specifically, among all subjects examined, there was a significantly greater thickness of EAT at the heart base region compared to near the apex region (1.016 ± 0.30 cm vs 0.727 ± 0.23 cm, *p* < 0.001). This discrepancy in results could be attributed to numerous large vessels present at the heart base area, which contains more gaps and inflection depressions than its superficially smoother proximal apical counterpart where adipose tissue tends to accumulate.

A Pd value is a straightforward and non-invasive electrocardiographic marker that reflects the extent of variation in the duration of overall atrial depolarization process. Previous studies have demonstrated the ability of Pd values to effectively differentiate patients with idiopathic paroxysmal AF from healthy controls, making it an important predictor for idiopathic AF [13]. The increase in Pd value primarily manifests as uneven extension of P-wave duration. Cardiac electrophysiological analysis in such patients revealed significant differences in conduction time and excitability of cardiac electrical signals compared to individuals with normal P-waves [14]. In our study, we observed a strong correlation between EAT thickness measured by echocardiography and Pd value, indicating that thickening of EAT leads to increased variability in P-wave duration. This suggests that the paracrine effect exerted by EAT and mechanical compression on atrial muscle may contribute to enhanced variability in atrial electrical conduction, ultimately leading to AF occurrence.

Our findings revealed no statistically significant difference in EAT thickness near the apex of the heart between the PAF group and persistent AF group (*p* = 0.181). However, there was still a notable statistical difference observed in EAT thickness near the heart base region; specifically, EAT thickness near the base was significantly higher among patients with persistent AF compared to those with paroxysmal AF. These results suggest a close association between EAT near the base region and both onset and duration of AF episodes. We speculate that local paracrine effects on left atrial myocardium may be more pronounced due to closer proximity between EAT near the base region and adjacent atrium. After conducting a comprehensive analysis of multiple potential clinical risk factors, it was confirmed that the thickness of EAT near the heart base independently contributed to an increased risk of AF (OR = 13.673, 95% CI 2.819~66.318, *p* = 0.01), which is consistent with previous studies. However, contrary to prior research findings, this study revealed that EAT thickness near the apex did not pose a significant risk for AF occurrence, suggesting an inconsistent relationship between epicardial fat in different locations and the development of AF. Previous investigations have demonstrated a close association between the amount of epicardial fat and pulmonary vein refractory period [15]. Specifically, greater thickness of EAT near the heart base corresponds to higher absolute fat content and increased secretion of active substances. Importantly, this site is adjacent to both the atrium and pulmonary veins, which facilitates direct interaction between secreted substances from adipose tissue and these cardiac structures; thus promoting electrical remodeling within them and ultimately leading to AF.

From the perspective of cardiac anatomy, there is no distinct fascial tissue separating EAT from the myocardium, and EAT is in direct proximity to the myocardium [16, 17, 18]. While it can act as a cushion for surrounding tissues to mitigate the strong diastolic movement of the myocardium and reduce mechanical impact on coronary arteries and their branches, excessive epicardial fat not only occupies space within the pericardium but also compresses the heart. Furthermore, excessive epicardial fat weakens the tightness and uniformity of connections between atrial muscles, resulting in heterogeneous conduction of electrical signals and increased anisotropy of cardiac conduction [19]. Based on these principles, the highly significant correlation between EAT thickness and Pdmay indicate that thickening of EAT induces atrial electrical remodeling leading to heterogeneity and anisotropy in cardiac electrical conduction, ultimately affecting changes in cardiac conduction duration and excitability.

The feasibility of utilizing EAT to predict the occurrence of AF has gained increasing support from some scholars, with demonstrated high accuracy [20]. However, in most studies, EAT measurement techniques involve cardiac magnetic resonance imaging (MRI) and computer tomography (CT), which are costly and challenging for widespread use. In contrast, echocardiography is more accessible, cost-effective, and conducive to clinical adoption [21]. In this study, two-dimensional images from the parasternal long axis of the left ventricle, a commonly used echocardiography practice, were chosen for observation. EAT concentration in the atrioventricular sulcus and ventricular sulcus was noted, consistent with prior research results. ROC analysis indicated that the area under the curve for EAT near the heart apex and EAT near the heart base in determining AF occurrence was 0.665 and 0.723, respectively. This suggests that while EAT thickness at both sites could predict AF, EAT thickness near the heart base exhibited better diagnostic efficiency, specificity, and sensitivity. Therefore, measuring EAT thickness near the base using the parasternal long axis section of the left ventricle is deemed helpful in easily identifying individuals prone to AF.

## 5. Conclusions

EATthickness, particularly near the heart base, as measured by echocardiography, demonstrates a close association with ECG P-wave dispersion. Notably, EAT thickness near the heart base is significantly linked to the occurrence of AF. It can aid in identifying individuals predisposed to AF to a certain extent, and its predictive value surpasses that of EAT thickness near the heart apex.

## Data Availability

Part of the dataset from this study has been presented in the paper, and readers could contact the corresponding author if they require the full dataset.

## References

[1] Hindricks G, Potpara T, Dagres N, Arbelo E, Bax JJ, Blomström-Lundqvist C (2021). 2020 ESC Guidelines for the diagnosis and management of atrial fibrillation developed in collaboration with the European Association for Cardio-Thoracic Surgery (EACTS): The Task Force for the diagnosis and management of atrial fibrillation of the European Society of Cardiology (ESC) Developed with the special contribution of the European Heart Rhythm Association (EHRA) of the ESC. *European Heart Journal*.

[2] Zain S, Shamshad T, Kabir A, Khan AA (2023). Epicardial Adipose Tissue and Development of Atrial Fibrillation (AFIB) and Heart Failure With Preserved Ejection Fraction (HFpEF). *Cureus*.

[3] Al-Makhamreh HK, Toubasi AA, Al-Harasis LM, Albustanji FH, Al-Sayegh TN, Al-Harasis SM (2023). Pericardial fat and cardiovascular diseases: A systematic review and meta-analysis. *Journal of Evidence-Based Medicine*.

[4] Iacobellis G (2023). Epicardial fat links obesity to cardiovascular diseases. *Progress in Cardiovascular Diseases*.

[5] Mant J, Fitzmaurice DA, Hobbs FDR, Jowett S, Murray ET, Holder R (2007). Accuracy of diagnosing atrial fibrillation on electrocardiogram by primary care practitioners and interpretative diagnostic software: analysis of data from screening for atrial fibrillation in the elderly (SAFE) trial. *British Medical Journal*.

[6] Pezzuto S, Gharaviri A, Schotten U, Potse M, Conte G, Caputo ML (2018). Beat-to-beat P-wave morphological variability in patients with paroxysmal atrial fibrillation: an in silico study. *Europace*.

[7] Baman JR, Passman RS (2021). Atrial Fibrillation. *JAMA*.

[8] Iacobellis G (2022). Epicardial adipose tissue in contemporary cardiology. *Nature Reviews. Cardiology*.

[9] Couselo-Seijas M, Rodríguez-Mañero M, González-Juanatey JR, Eiras S (2021). Updates on epicardial adipose tissue mechanisms on atrial fibrillation. *Obesity Reviews*.

[10] Abe I, Teshima Y, Kondo H, Kaku H, Kira S, Ikebe Y (2018). Association of fibrotic remodeling and cytokines/chemokines content in epicardial adipose tissue with atrial myocardial fibrosis in patients with atrial fibrillation. *Heart Rhythm*.

[11] van Rosendael AR, Dimitriu-Leen AC, van Rosendael PJ, Leung M, Smit JM, Saraste A (2017). Association Between Posterior Left Atrial Adipose Tissue Mass and Atrial Fibrillation. *Circulation. Arrhythmia and Electrophysiology*.

[12] Thanassoulis G, Massaro JM, O’Donnell CJ, Hoffmann U, Levy D, Ellinor PT (2010). Pericardial fat is associated with prevalent atrial fibrillation: the Framingham Heart Study. *Circulation. Arrhythmia and Electrophysiology*.

[13] Dilaveris PE, Gialafos EJ, Sideris SK, Theopistou AM, Andrikopoulos GK, Kyriakidis M (1998). Simple electrocardiographic markers for the prediction of paroxysmal idiopathic atrial fibrillation. *American Heart Journal*.

[14] Unkell M, Marinov M, Wolff PS, Radziejewska J, Mercik JS, Gajek J (2020). P wave duration in paroxysmal and persistent atrial fibrillation. *Advances in Clinical and Experimental Medicine*.

[15] Munger TM, Dong YX, Masaki M, Oh JK, Mankad SV, Borlaug BA (2012). Electrophysiological and hemodynamic characteristics associated with obesity in patients with atrial fibrillation. *Journal of the American College of Cardiology*.

[16] Marchington JM, Mattacks CA, Pond CM (1989). Adipose tissue in the mammalian heart and pericardium: structure, foetal development and biochemical properties. *Comparative Biochemistry and Physiology. B, Comparative Biochemistry*.

[17] Meenakshi K, Rajendran M, Srikumar S, Chidambaram S (2016). Epicardial fat thickness: A surrogate marker of coronary artery disease - Assessment by echocardiography. *Indian Heart Journal*.

[18] Villasante Fricke AC, Iacobellis G (2019). Epicardial Adipose Tissue: Clinical Biomarker of Cardio-Metabolic Risk. *International Journal of Molecular Sciences*.

[19] Zhou M, Wang H, Chen J, Zhao L (2020). Epicardial adipose tissue and atrial fibrillation: Possible mechanisms, potential therapies, and future directions. *Pacing and Clinical Electrophysiology*.

[20] Iacobellis G, Willens HJ (2009). Echocardiographic epicardial fat: a review of research and clinical applications. *Journal of the American Society of Echocardiography*.

[21] Beyer C, Tokarska L, Stühlinger M, Feuchtner G, Hintringer F, Honold S (2021). Structural Cardiac Remodeling in Atrial Fibrillation. *JACC Cardiovasc Imaging*.

